# Associations between Elemental Metabolic Dynamics and Default Mode Network Functional Connectivity Are Altered in Autism

**DOI:** 10.3390/jcm12031022

**Published:** 2023-01-28

**Authors:** Paul Curtin, Janina Neufeld, Austen Curtin, Christine Austin, Johan Isaksson, Karl Lundin Remnelius, Hjalmar Nobel Norrman, Manish Arora, Sven Bölte

**Affiliations:** 1 Linus Biotechnology Inc., New York, NY 10013, USA; 2 Center of Neurodevelopmental Disorders (KIND), Centre for Psychiatry Research, Department of Women’s and Children’s Health, Karolinska Institutet & Stockholm Health Care Services, Region Stockholm, 11330 Stockholm, Sweden; 3 Swedish Collegium for Advanced Study, 75238 Uppsala, Sweden; 4 Environmental Medicine and Public Health, Mount Sinai School of Medicine, New York, NY 10029, USA; 5 Department of Medical Sciences, Child and Adolescent Psychiatry Unit, Uppsala University, 75185 Uppsala, Sweden; 6 Curtin Autism Research Group, Curtin School of Allied Health, Curtin University, Perth, WA 6102, Australia; 7 Child and Adolescent Psychiatry, Stockholm Health Care Services, Region Stockholm, 11861 Stockholm, Sweden

**Keywords:** autism, default mode network, dynamical systems, environmental exposures, exposomics, metal exposures

## Abstract

Autism is a neurodevelopmental condition associated with atypical social communication, cognitive, and sensory faculties. Recent advances in exposure biology suggest that biomarkers of elemental uptake and metabolism measured in hair samples can yield an effective signal predictive of autism diagnosis. Here, we investigated if elemental biomarkers in hair were associated with functional connectivity in regions of the default mode network (DMN) previously linked to autism. In a study sample which included twin pairs with concordant and discordant diagnoses for autism, our analysis of hair samples and neuroimaging data supported two general findings. First, independent of autism diagnosis, we found a broad pattern of association between elemental biomarkers and functional connectivity in the DMN, which primarily involved dynamics in zinc metabolism. Second, we found that associations between the DMN and elemental biomarkers, particularly involving phosphorus, calcium, manganese, and magnesium, differed significantly in autistic participants from control participants. In sum, these findings suggest that functional dynamics in elemental metabolism relate broadly to persistent patterns of functional connectivity in the DMN, and that these associations are altered in the emergence of autism.

## 1. Introduction

Autism is a pervasive neurodevelopmental condition thought to emerge from a confluence of genetic and environmental factors [1,2,3]. The global prevalence of autism is 1 to 3% with a male to female ratio of 3:1 [4,5]. While autism today is exclusively defined and assessed behaviorally, the search for biomarkers and related underlying mechanisms is critical to earlier, more objective, and more reliable diagnoses [6]. There have been two promising lines of research towards this goal, which include chemical and functional biomarkers identified on different timescales. Chemical biomarkers, including essential and toxic metals, involve time-dependent mechanisms on a time-scale of weeks to months [7,8,9,10]. Functional biomarkers, particularly involving measures of electrophysiological dynamics and/or functional connectivity, emerge on a timescale of seconds to minutes [11]. Here, we explore the relationship between chemical and functional processes to determine if chemical dysregulation relates to the underlying neural mechanisms implicated in autism.

Generally, atypical metals regulation is widely implicated in altered neurodevelopment [12,13,14,15]; this topic has been addressed in several recent systemic reviews [2,12,16,17]. More recently, atypical metal regulation has been specifically implicated in autism, involving either time-dependent vulnerabilities, e.g., critical windows of exposure, over the span of weeks [18]; or, a persistent atypical regulation of metal intake and metabolism [7]. Previously, Curtin et al. [7] showed that an atypical regulation of metal metabolism is predictive of autism in multiple independent populations. With laser-assisted mass spectrometry, Curtin et al. [7] measured metals along growth increments (like tree rings) in shed baby teeth and generated longitudinal profiles of metals exposure from prenatal development to 7 years of age. These profiles allowed Curtin et al. [7] to characterize the dynamic properties of elemental metabolism; particularly, zinc and copper biomarkers were predictive of autism. Austin et al. [9] recently expanded this work to hair, showing that metal biomarkers (including zinc and copper) can be used to predict autism in multiple populations from the ages of 0 to 18 years.

Mechanistic processes are also a major focus of electrophysiological and neuroimaging research, which operate on timescales ranging from milliseconds to minutes. Resting state (RS) functional magnetic resonance imaging (fMRI), in particular, has been used to characterize atypical regulation of spontaneous (or task-free) activity in neurodevelopmental conditions [19]. In autism, a primary focus of RS activity has involved the default mode network (DMN), a prominent RS network where atypical DMN connectivity has been suggested to be a core feature of autism [20,21,22,23,24]. DMN functional connectivity is more commonly reported to be decreased in adults and adolescents with autism [25,26] although autistic children might also present with increased DMN connectivity [27].

Functional connectivity data in general, and DMN-related analyses specifically, have traditionally been analyzed with correlation-based analyses. Curtin et al. [11] recently developed an approach for predictive classification of autism from the analysis of DMN data using recurrence quantification analysis (RQA) and cross-recurrence quantification analysis (CRQA), which present a non-linear alternative to traditional correlation-based analyses. In contrast to correlation-based analyses, the application of RQA yields multiple quantitative metrics descriptive of periodicity, stability, and complexity within a given signal, which CRQA extends to the analysis of paired signals. This work identified the dysregulation of dynamics across the whole DMN network, and more specifically involving two core hub regions of the DMN, the right lateral parietal lobe (LP_R_) and medial prefrontal cortex (MPFC) [11]. More specifically, Curtin et al. [11] found that the determinism of these signals, which indicates the prevalence of periodic dynamics, was dysregulated in these regions in autism. Related work has likewise examined measures which characterize signal stability, such as laminarity, in the DMN of autistic individuals [28].

In the present study, we sought to investigate connections between biochemical biomarkers and functional measures of autism. Building on our prior work, which identified four target functional biomarkers in DMN functional dynamics, we utilized chemical biomarkers generated from the analysis of hair samples to investigate two general hypotheses. First, we tested if chemical dynamics in hair relate generally to functional dynamics in the DMN. Next, we tested if these associations differed in autistic individuals. Our results place new emphasis on the importance of elemental intake and metabolism on typical neurodevelopment and implicate a functional dependence between chemical and neural mechanisms underlying autism.

## 2. Materials and Methods

### 2.1. Participants

Participants (*n* = 124) in this study were recruited as part of the Roots of Autism and ADHD Twin Study in Sweden (RATSS) [29]. These participants have been described elsewhere in detail [11]. Briefly, twin pairs where one or both twins showed traits of autism, were recruited to RATSS, where they underwent diagnostic assessment for autism, ADHD, and other mental health conditions, performed by experienced clinicians. Additionally, twin pairs where no twin displayed elevated autistic traits were recruited as controls. Participants with profound intellectual disability (IQ < 35), serious psychiatric (e.g., paranoid schizophrenia) or neurological conditions (e.g., intractable epilepsy), or any genetic syndrome (e.g., fragile X) were excluded from recruitment. Additionally, participants with metallic implants, brain surgery, or claustrophobia, were excluded from neuroimaging assessment. Participant demographics are summarized in Table 1. The Regional Swedish Ethical Review Board approved all study protocols, and participants, or their caregivers, provided informed consent.

### 2.2. Neuroimaging

**Figure 1:**
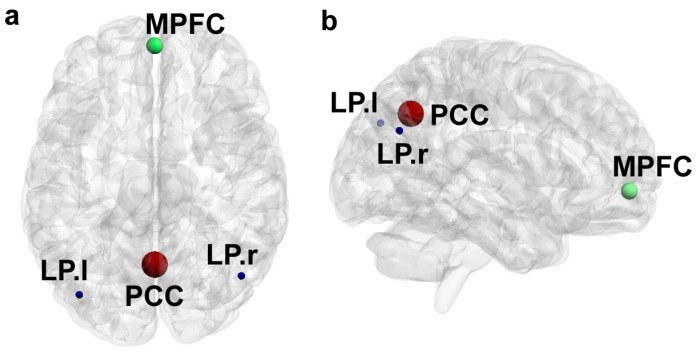
Glass brain schematic of the default mode network (DMN) endpoints (**a**) Dorsal view of the medial prefrontal cortex (MPFC, green), posterior cingulate cortex (PCC, red), left lateral parietal (LP.l, blue), and right lateral parietal (LP.r, blue) regions. (**b**) Lateral view of the MPFC (green), PCC (red), left lateral parietal (blue), and right lateral parietal (blue) regions.

Scanning methods were previously described [11]. Briefly, participants completed a 5 to 7 min pre-scanning training session in a mock scanner, followed by a ~50 min MRI session in a 3 Tesla MR750 GE-scanner (GE Healthcare, Chicago, IL, USA). As described previously [11], the scanning session included a ~5 min T1-weighted Spoiled Gradient Echo high resolution anatomical scan (176 slices, TR = 8.2 s, FOV = 240 mm) and a 10 min resting state (RS) T2*-weighted Echo Planar Imaging Scan (45 slices, TR = 3 s, 205 volumes, FOV = 288 mm, matrix size = 96 × 96). A white cross on black background was presented during the RS scan; participants were instructed to look at the cross throughout the RS functional MRI run. Data were preprocessed with the default direct normalization to MNI-space pipeline in CONN [Matlab v2017b (Mathworks), https://www.nitrc.org/projects/conn, accessed on 25 March 2019] which includes the following processes: (1) motion estimation and correction, (2) slice-timing correction, (3) outlier detection [Artifact Detection Tools (ART)-based scrubbing], (4) normalization to MNI (Montreal Neurological Institute) space, and (5) smoothing (8mm Gaussian kernel). Slices were processed in interleaved bottom-up order, and outlier detection was set to intermediate (97th percentile in normative sample). Data were denoised with linear regression by correcting for the effects of head motion, physiology, and other artifacts. Finally, signals were bandpass filtered (0.008–0.090 Hz). We defined the DMN ROIs based on anatomical brain regions by co-registration with MNI space using the Harvard-Oxford brain atlas (see Figure 1 for glass brain schematic) [30,31,32,33].

### 2.3. Hair Analysis

A single hair strand was selected from each participant for elemental analysis by la-ser ablation-inductively coupled plasma-mass spectrometry (LA-ICP-MS). To remove possible surface contamination, hairs were washed in a solution of 1% Triton X-100 and ultra-pure water (18.2 MΩcm^−1^) with sonication for 1 min, then rinsed with ultra-pure water and dried in an oven at 60 °C overnight. Double sided tape was used to mount dried hairs on glass microscope slides. Laser ablation was performed under a helium atmosphere using a New Wave Research NWR-193 (ESI, Beaverton, OR, USA) laser equipped with a 193 nm ArF excimer laser. The ablated material was transferred to an Agilent Technologies 8800 triple-quad ICP-MS (Agilent Technologies, Santa Clara, CA, USA) via a stream of helium, mixed with argon before the plasma. LA-ICP-MS sensitivity (maximum analyte ion counts), oxide formation (232Th16O+/232Th+, <0.3%) and fractionation (232Th+/238U+, 100 ± 5%) was tuned daily using NIST SRM 612 (trace elements in glass). As a further precaution against possible surface contamination, hairs were first ablated at low laser energy (0.27–0.32 Jcm^−2^) to remove the surface layer. Following the pre-ablation scan, a higher energy (0.50–0.55 Jcm^−2^) scan was then performed and element signal intensity measured by the ICP-MS. A distance representing approximately 1 month of growth (10 mm, over 650 sampling points) was ablated along each hair from the end closest to the scalp to the tip. Element intensities were normalized to sulfur (e.g., 66Zn:34S) to control for tissue density variations within a hair and between samples.

### 2.4. Feature Engineering

Blood-oxygen-level-dependent (BOLD) signals extracted from the right lateral parietal lobe (LP_R_), medial prefrontal cortex (MPFC), and the averaged BOLD signal across all regions of the DMN (LP_L_, LP_R_, MPFC, and posterior cingulate cortex; see [11]) were subjected to recurrence quantification analysis (RQA) or cross-recurrence quantification analysis (CRQA) to extract multiple features. Based on previously reported findings [11], four features were derived from application of RQA and CRQA. These included determinism in the overall (averaged) DMN BOLD signal, and determinism in LP_R_ BOLD signal. In the application of CRQA, we utilized determinism in MPFC: LPR signals, and determinism in LP_R_: LP_L_ signals. These features were selected based on their established association with autism [11].

For the analysis of chemical signals, based on [9], we likewise applied RQA/CRQA to elemental time-series measured in hair, and to pairwise combinations of elemental time series. We generated features from 15 elemental time series in hair, including Aluminum (Al), Arsenic (As), Barium (Ba), Bismuth (Bi), Calcium (Ca), Copper (Cu), Lithium (Li), Magnesium (Mg), Manganese (Mn), Phosphorous (P), Lead (Pb), Sulfur (S), Tin (Sn), Strontium (Sr), and Zinc (Zn). For each elemental time series (e.g., Zn) and paired interactions between them (e.g., Zn and Ba), a recurrence matrix or cross-recurrence matrix, respectively, was generated to reconstruct underlying signal dynamics [34,35]. Unlike the application of RQA/CRQA to BOLD signals, which was intentionally limited to features already linked to autism, we generated a broader array of dynamical features in our analysis of chemical signals. These included: determinism, entropy, and mean diagonal length, which measure the prevalence, complexity, and duration of periodic processes, respectively; additionally, we measured mean recurrence time, which captures the mean frequency of periodic processes. We also measured laminarity, trapping time, and (vertical) entropy, which likewise capture the prevalence, duration, and complexity of stable states in a given time-series; that is, periods where variability in the series is minimal. In sum, given 15 time series and associated pairwise interactions, and 7 features derived from each such analysis, we considered in total 1470 chemical signal features in total in connection with the four neuroimaging measures.

Details on the implementation of RQA/CRQA analyses are provided in [9,11]. Briefly, RQA/CRQA requires first the derivation of associated recurrence plots as described in our prior studies s [7,8,10,36]. Recurrence plots were created by minimizing mutual information (delay, τ) and false-nearest neighbor (embedding dimension, m) parameters. Threshold functions, ε, were constrained to yield 10% recurrence rates to facilitate cross-subject comparisons. All C/RQA analyses were performed with the Dynamical Systems library (https://juliadynamics.github.io/DynamicalSystems.jl/latest/, accessed on 24 November 2022) in Julia [37].

### 2.5. Statistical Analysis

**Figure 2:**
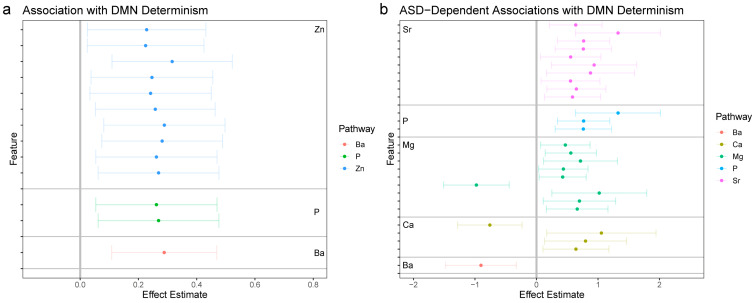
Associations between elemental dynamics and DMN functional connectivity. (**a**) Left panel shows effect estimates for associations between elemental features and determinism in the average DMN signal; only features with significant associations following FDR-adjustment for multiple comparisons are shown. (**b**) Right panel shows effect estimates for the interaction between autism and features of elemental dynamics; features with significant associations following FDR-adjustment for multiple comparisons are shown. The magnitude of effect estimates in essence depicts the difference in a given feature between participants with autism and control participants.

**Figure 3:**
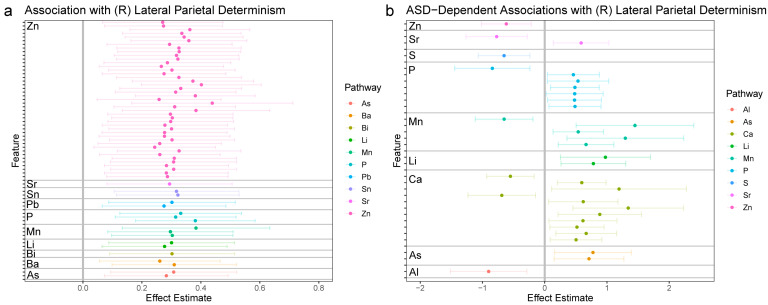
Associations between elemental dynamics and right lateral parietal lobe. (**a**) Left panel shows effect estimates for associations between elemental features and determinism in the right lateral parietal lobe BOLD signal; only features with significant associations following FDR-adjustment for multiple comparisons are shown. (**b**) Right panel shows effect estimates for the interaction between autism and features of elemental dynamics; features with significant associations following FDR-adjustment for multiple comparisons are shown. The magnitude of effect estimates in essence depicts the difference in a given feature between participants with autism and control participants.

**Figure 4:**
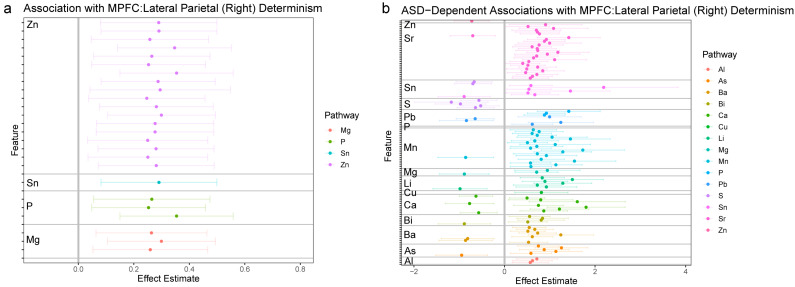
Associations between elemental dynamics and synchronization between medial prefrontal cortex and right lateral parietal lobe. (**a**) Left panel shows effect estimates for associations between elemental features and determinism in the LP_R_ and MPFC BOLD signals; only features with significant associations following FDR-adjustment for multiple comparisons are shown. (**b**) Right panel shows effect estimates for the interaction between autism and features of elemental dynamics; features with significant associations following FDR-adjustment for multiple comparisons are shown. The magnitude of effect estimates in essence depicts the difference in a given feature between participants with autism and control participants.

**Figure 5:**
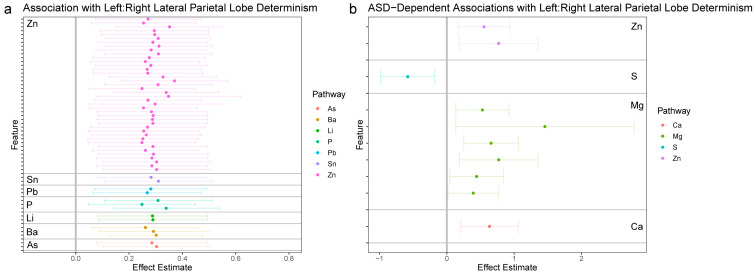
Associations between elemental dynamics and synchronization between left and right lateral parietal lobes. (**a**) Left panel shows effect estimates for associations between elemental features and determinism in the LP_L_ and LP_R_ BOLD signals; only features with significant associations following FDR-adjustment for multiple comparisons are shown. (**b**) Right panel shows effect estimates for the interaction between autism and features of elemental dynamics; features with significant associations following FDR-adjustment for multiple comparisons are shown. The magnitude of effect estimates in essence depicts the difference in a feature between participants with autism and control participants.

The fundamental aims of the statistical analysis were to link the 1470 features derived from chemical analysis of hair samples to the four functional connectivity outcomes derived from the neuroimaging analysis. This was achieved through a strategy akin to a GWAS-type analysis, wherein each feature was tested for association with each of the four outcomes in a discrete linear model. Because some samples were derived from twin samples, mixed linear models were used to account for relatedness among twins through the inclusion of a random factor for twin pair. All models were likewise adjusted for sex, zygosity, mean motion in the scanner, and age of the participant at the time of the scan. All tests were subjected to false discovery rate (FDR)-adjustment for multiple comparisons; to minimize Type II errors, these were stratified according to the elemental pathway investigated. Relative to the goals of the study, each model included two parameters to test the primary research hypothesis. First, a simple main effect was included to test for the association between a given chemical feature and a neural outcome. Second, to test if these effects differed in individuals diagnosed with autism compared to non-autistic controls, a multiplicate parameter (interaction effect) was included in the model. Figure 2, Figure 3, Figure 4 and Figure 5 provide effect estimates associated with main effects and multiplicative effects, respectively, in cases where effects were still significant after multiple comparison adjustment.

## 3. Results

### 3.1. Sample Characteristics

Hair samples and neuroimaging data were collected for 124 participants in total; Table 1 provides a full breakdown of participant demographic characteristics. The percentage of male to female participants did not differ significantly (β = 0.13, *p* = 0.47); however, male participants were approximately 1.5 years younger than female participants (β = −1.58, *p* = 0.03) at the time of the fMRI scan. There was no significant difference in the age of participants with autism and controls (β = 0.39, *p* = 0.64).

For each of 15 elements measured in every participant’s hair sample, and for each pairwise elemental interaction, we generated seven descriptive metrics to characterize temporal dynamics in elemental uptake and metabolism, yielding 1470 discrete chemical signal features. We linked these to four endpoints in the DMN, which were previously linked to autism. These included periodicity in the averaged DMN signal, and the (1) right lateral parietal (LPR) region; and synchronization of periodic processes between the (2) medial prefrontal cortex (MPFC) and (3) right lateral parietal (LPR), and the (4) left lateral parietal (LPL) and LPR (see Figure 1 for glass brain schematic).

### 3.2. Associations between Elemental and Functional Dynamics

#### 3.2.1. DMN-Wide Associations

We began by testing for associations between elemental biomarkers and periodicity in the averaged BOLD signal across all regions of the DMN. Figure 2a (left panel) shows associations (effect estimates, with confidence intervals) for a broad array of elemental features which were significantly associated with DMN periodicity after FDR-adjustment for multiple comparisons.

Two particularly notable patterns emerged from this analysis. First, associations which survived adjustment for multiple comparison were uniformly positive, indicating that increased periodicity in elemental metabolism is linked with increased periodicity in the functional dynamics. Second, although features of phosphorus (P) and barium (Ba) metabolism were associated with DMN dynamics, zinc (Zn) dynamics were notably more prominent, in that 10 distinct feature-association’s survived multiple comparison adjustment as compared to two (phosphorus) or one (barium) for other elements. We next estimated the effect that autism had on a given feature’s association with DMN dynamics; to determine, in other words, which associations differed in autistic individuals vs. controls. Figure 2b (right panel) presents effect estimates and associated confidence intervals for the interaction between autism and a given elemental feature in relation to DMN functional dynamics.

We found a pattern of broad dysregulation involving strontium (Sr), phosphorus (P), magnesium (Mg), calcium (Ca), and barium (Ba). These associations were mostly positive, indicating stronger positive associations in autistic individuals than controls, but also included some negative effects, which indicate a negative association in autism relative to a positive association in controls.

#### 3.2.2. Right Lateral Parietal (LP_R_)

We next extended this analytical approach to functional dynamics in subregions of the DMN previously linked to autism. In Figure 3a (left panel), we show effect estimates relating elemental features to periodicity in the right lateral parietal lobe (LP_R_). As in the DMN, overall, we identified a broad pattern of associations between elemental dynamics and functional dynamics in the LP_R_. These associations were uniformly positive, and primarily driven by zinc. In Figure 3b (right panel), we show associations that differed in autistic individuals relative to controls. The most prominent elemental pathways involved in these effects were phosphorus (P), calcium (Ca), and manganese (Mn), though other elements (Zn, Sr, S, Li, As, and Al) were also involved. These effects were primarily positive, indicating that in autistic individuals, associations between elemental features and neural dynamics were significantly stronger than in controls.

#### 3.2.3. MPFC: LP_R_ Synchronization

In Figure 4a (left panel), we highlight associations between features of elemental dynamics and functional connectivity between the medial prefrontal cortex (MPFC) and the right lateral parietal (LP_R_), as measured in Curtin et al. [8] via the quantification of CRQA determinism. As in the analysis of periodicity in the overall DMN, and within the LP_R_, specifically, we found again a pattern of positive associations involving multiple elements, primarily dominated by zinc.

In considering the effects of autism on associations between elemental metabolism and MPFC: LP_R_ connectivity, we found a systemic pattern of effects that involved every elemental pathway (Figure 4b, right panel). These effects were nonetheless primarily associated with a few specific elements, particularly strontium (Sr) and manganese (Mn), with notable involvement of Sn, Pb, Li, Ca, Ba, and As, as well. As in other autism-dependent effects, we noted these associations were primarily positive, indicative of stronger associations in autistic individuals, but also involved some negative effects, which indicate weaker or negative associations in autistic individuals.

#### 3.2.4. LP_L_ and LP_R_ Synchronization

In Figure 5a (left panel) we highlight associations between elemental dynamics and synchronization between the left lateral parietal lobe (LP_L_) and right lateral parietal lobe (LP_R_). Consistent with other regions of the DMN, and with analysis of the averaged DMN signal, we identified several elemental dynamics relating to LP_L_: LP_R_ dynamics, but these effects were overwhelmingly dominated by zinc. Likewise, as in all other analyses, we found these effects were uniformly positive, indicating that increased periodicity in elemental dynamics was associated with increased synchronization in periodic dynamics in the brain.

In contrast to other DMN subregions, we found relatively few effects survived multiple comparison when testing for differences between autistic individuals and controls. Figure 5b shows the relatively few effects which survived multiple comparison adjustment, primarily involving magnesium metabolism, with some involvement of zinc, sulfur, and calcium.

## 4. Discussion

We tested two general hypotheses relating elemental dynamics to functional connectivity in the default mode network (DMN). First, we tested if functional dynamics in the intake and metabolism of 15 different elements related to periodicity and synchronization in the DMN, generally, in a sample which included neurotypical and autistic individuals. Our results indicate that a broad array of features in elemental metabolism, particularly involving zinc, relate to functional dynamics in the DMN. Second, we tested if associations between elemental dynamics and DMN connectivity were different in autistic individuals when compared with controls. We identified a number of elemental pathways, particularly involving magnesium, manganese, calcium, and phosphorus, where the association between metabolic dynamics and neural connectivity was different in autistic individuals. In sum, our results broadly indicate that metabolic dynamics involved in the intake of essential elements relate to functional dynamics in the DMN, and these associations are altered in autistic individuals.

The neural endpoints for the current study were selected based on our previous findings [11] using recurrence quantification analysis (RQA) of BOLD signals (averaged DMN, or right lateral parietal), or cross-recurrence quantification analysis (CRQA) of paired BOLD signals (left: right lateral parietal, and medial prefrontal cortex: right lateral parietal), respectively. These findings align with a broad range of studies which have consistently linked autism with dysregulation of DMN connectivity [21,23,24,26,27,28]. Importantly, the present results provide a new mechanistic explanation to help explain this pattern of findings, in that we show metal dysregulation relates to functional dynamics in the DMN, and that this relationship is systemically altered in autism.

It is nonetheless likely that by selectively focusing on regions of interest (ROIs) in the DMN that are already linked to autism, this study may overlook other regions of interest potentially impacted by metals dysregulation. Recent studies examining elemental metabolic dynamics in relation to autism [7,9] have consistently linked copper dynamics to ASD diagnosis, for example, whereas here we found that associations between copper metabolism and DMN connectivity were relatively unperturbed in individuals with autism. Likely, this reflects the relatively narrow focus of this study, which exclusively examined functionality in the DMN, and suggests that future studies should focus on the role of elemental dynamics in connectivity outside the DMN. Indeed, prior studies have shown that the putamen and globus pallidus, for example, were associated with excess manganese accumulation [38,39,40,41]. Similarly, lead exposure impacts cross-hemispheric and long-range connectivity [42]. Accordingly, given the sensitivity of regions outside the DMN to metal dysregulation, future studies linking metals dysregulation to functional dynamics should consider a data-driven exploration utilizing a broader scope of ROIs.

The elemental pathways targeted in this study were likewise selected on the basis of prior studies, and in general, the findings are in agreement with prior literature. For example, zinc, the element most prominently linked to DMN functional dynamics in the present study, was previously linked to neonatal neurodevelopment [43,44,45,46]. Zinc deficiency can yield atypically regulated cell cycle pathways critical to neurogenesis, neuronal migration, differentiation, and apoptosis [44]; and, zinc supplementation has, in some clinical trials [43,46], supported typical behavioral development. Particularly relevant to the present finding, Takeuchi et al. [47] found that zinc concentrations measured in hair related to DMN connectivity. Here, we show that dynamics in zinc metabolism (as opposed raw measures of concentration) also impact functional dynamics in the DMN.

The elemental pathways where we observed atypical associations with DMN signaling in autistic individuals are likewise well-established mediators of neurodevelopment and synaptic signaling. Calcium, magnesium, manganese, and phosphorus, the most prominently altered pathways in autism, are each critical either to long term structural development, or to synaptic transmission in the short term, and as such are tightly regulated homeostatic mechanisms [48,49,50,51,52,53,54,55]. Notably, prior studies have also linked atypically regulated manganese and zinc dynamics to autism [18]. Consistent with these findings and those reported in the present study, Fiore at el. [56] linked zinc levels measured in hair to the severity of autism symptoms. Additionally, similar to our results, they likewise identified a range of other elements including lead, manganese, and molybdenum linked to cognitive functioning in individuals with autism. Our results, which indicate altered linkages between homeostatic dynamics in these elements and DMN functional dynamics in autism, support and extend these findings to a broader array of elements.

Conclusions drawn from the present study must nonetheless be tempered by limitations implicit in the design. First, notably, the available sample size (*n* = 124) was modest and was largely drawn from a fairly homogenous population of Swedish descent. As such, generalization of these results may be limited, particularly to environments where elemental exposures may be markedly different. As well, the targeted nature of this study, which focused on linkages neural and chemical endpoints assessed in prior studies, likely ignores other potentially important mechanisms relevant to autism.

## 5. Conclusions

In sum, this study offers important evidence that dynamics in elemental intake and metabolism relate to functional connectivity in the DMN, and these relationships are altered in autistic individuals. These results support prior literature which links zinc intake and metabolism to typical neurodevelopment, and suggests the role of multiple essential elements, including calcium, manganese, phosphorus, and magnesium, in regulating DMN connectivity may be altered in autistic individuals. Future studies should extend these findings with a broader exploration of linkages between elemental dynamics and structural and functional neurodevelopment, both in neurotypical and neurodivergent individuals.

## 6. Patents

Patents resulting from the hair analysis in this work include “Systems and methods for diagnostics for biological disorders associated with periodic variations in metal metabolism [57].

## Figures and Tables

**Table 1:** Participant demographics. ^1^ MZ and DZ refer to monozygotic and dizygotic twins.

	Total	Autism	Control	Zygosity (MZ, DZ) ^1^	Age (Mean ± SD)
Overall	124	30	94	81 MZ, 43 DZ	15.32 ± 4.02
Male	66	17	49	44 MZ, 22 DZ	14.59 ± 2.89
Female	58	13	45	37 MZ, 21 DZ	16.15 ± 4.91

## Data Availability

Data generated in this study may include personal health information, and as such, all reasonable requests must be evaluated by appropriate ethical review committees at the involved institutions.
